# Impact of nominal photon energies on normal tissue sparing in knowledge-based radiotherapy treatment planning for rectal cancer patients

**DOI:** 10.1371/journal.pone.0213271

**Published:** 2019-03-07

**Authors:** Yuliang Huang, Sha Li, Haizhen Yue, Meijiao Wang, Qiaoqiao Hu, Haiyang Wang, Tian Li, Chenguang Li, Hao Wu, Yibao Zhang

**Affiliations:** 1 Key Laboratory of Carcinogenesis and Translational Research (Ministry of Education/Beijing), Department of Radiation Oncology, Peking University Cancer Hospital & Institute, Beijing Cancer Hospital & Institute, Beijing, China; 2 Department of Medical Physics, Institute of Medical Humanities, Peking University, Beijing, China; North Shore Long Island Jewish Health System, UNITED STATES

## Abstract

The interactive adjustment of the optimization objectives during the treatment planning process has made it difficult to evaluate the impact of beam quality exclusively in radiotherapy. Without consensus in the published results, the arbitrary selection of photon energies increased the probability of suboptimal plans. This work aims to evaluate the dosimetric impact of various photon energies on the sparing of normal tissues by applying a preconfigured knowledge-based planning (RapidPlan) model to various clinically available photon energies for rectal cancer patients, based on model-generated optimization objectives, which provide a comparison basis with less human interference. A RapidPlan model based on 81 historical VMAT plans for pre-surgical rectal cancer patients using 10MV flattened beam (10X) was used to generate patient-specific objectives for the automated optimization of other 20 patients using 6X, 8X, 10X (reference), 6MV flattening-filter-free (6F) and 10F beams respectively on a TrueBeam accelerator. It was observed that flattened beams produced very comparable target dose coverage yet the conformity index using 6F and 10F were clinically unacceptable (>1.29). Therefore, dose to organs-at-risk (OARs) and normal tissues were only evaluated for flattened beams. RapidPlan-generated objectives for 6X and 8X beams can achieve comparable target dose coverage as that of 10X, yet the dose to normal tissues increased monotonically with decreased energies. Differences were statistically significant except femoral heads. From the radiological perspective of view, higher beam energy is still preferable for deep seated tumors, even if multiple field entries such as VMAT technique can accumulate enough dose to the target using lower energies, as reported in the literature. In conclusion, RapidPlan model configured for flattened beams cannot optimize un-flattened beams before adjusting the target objectives, yet works for flattened beams of other energies. For the investigated 10X, 8X and 6X photons, higher energies provide better normal tissue sparing.

## Introduction

The inherent photon beam characteristics of different energies, such as penetrating power and penumbra, have provided planners with more options[1], but the choices also vary subjectively among different centers. There have been a lot of dosimetric researches on the beam energies [2,3,4,5], but consensus can hardly be made providing contradictory literature reports. Coming to the era of knowledge-based planning, the impact of beam energies on the model configuration and applications remains unknown, and is worthy of more investigations.

Varian RapidPlan (Varian Medical Systems, Pala Alto, CA) uses Principal Component Analysis (PCA) regression to fit the correlation between the geometric features (including patient anatomy and beam geometry) and the historical ‘achievable’ dose distribution without using the actual beam energy as regression input at the training stage. When the trained model is used to predict DVHs for upcoming cases, Geometry-Based Expected Dose (GED) metric was used to estimate the achievable dose to a voxel by considering patient anatomy (such as distance from the targets surfaces), dose prescriptions, and field geometry (such as in- *vs*. out-of-field) [6,7] and beam energy, etc. According to the manufacturer[8], the total dose-distance value in voxel v (*ged*_*v*_) is defined as:

$$ged_{v}=\sum_{t=1}^{m}\delta t\times \sum_{f=1}^{n}C_{tv}\times \frac{e^{-\lambda_{f}\times d_{fv}}}{d_{fv}^{2}}$$ (1)

Where *m* is number of target levels, *δ*_*t*_ is incremental target level, *n* is number of fields, *C*_*TV*_ is a scaling factor of modulation, *d*_*fv*_ is Euclidean distance from field *f* to voxel *v*, and *λ*_*f*_ is a parameter depending on the nominal photon beam energy of field *f*. A sum of the voxels yields a predicted dose volume histogram (DVH) range that can be used to generate patient specific optimization objectives. RapidPlan generated different objectives for the same patient in an automatic and mathematical manner if various beam energies were selected as input to the model prediction.

Previous studies on the dosimetric effects of photon energies were vulnerable to potential bias of different optimization objectives[9], or limitations of planner experience [5, 10]. By introducing *λ*_*f*_, RapidPlan generates various energy-dependent and patient specific objectives without subjective manual iterative adjustment and inter-planner variability, hence provides a more objective comparison basis.

This study aims to evaluate the dosimetric impact of beam qualities on the sparing of normal tissues by applying a preconfigured RapidPlan model to various clinically available photon energies for rectal cancer patients.

## Methods

This retrospective, anonymous and computation-based study is approved by Medical Ethics Committee of Peking University Cancer Hospital & Institute with exemption of informed consent. All experiments were conducted on Varian RapidPlan knowledge-based treatment planning system V. 13.5 with appropriate anonymization.

### The RapidPlan DVH estimation model

A published RapidPlan model for pre-surgical rectal cancer patients was used in this study[11,12,13]. As a brief review, the model was trained with 81 historical plans that were contoured and planned following Li's study [14]. Dose-volume constraints for normal tissues were in accordance with RTOG 0822 protocols [15]. Attempts were also made to reduce the organ mean dose to minimize long-term toxicity associated with low-dose region [16,17,18]. Considering the large target volumes were relatively deep-seated, all historical plans were optimized with 10 MV flattened photon beams (10X) by experts. The robustness of the model has been validated on over 100 cases [11–13].

### Knowledge-based planning using various energies

On the Eclipse treatment planning system V13.5, the aforementioned RapidPlan model was applied to estimate the best achievable DVHs under five photon beam qualities from a Varian TrueBeam accelerator equipped with Millennium 120 multi-leaf collimator (MLC), including 6-MV flattened (6X), 6-MV flattening-filter-free mode (6F), 8X, 10X and 10F respectively, for 20 historical patients that were not included in the model library. Higher energies are not used at our center for the consideration of secondary neutron contamination [19], hence were not tested in this study. Without any human intervention, VMAT plans were optimized using the RapidPlan-generated patient-specific objectives [20], keeping the original beam geometries of the clinical plans unchanged. The prescription dose was 41.8Gy for PTV and 50.6Gy for PTV_boost_. The volume dose was calculated using analytical anisotropic algorithm (AAA). All plans were normalized to cover 95% target volume with 100% prescription dose before comparison.

### Dosimetric assessment and statistical analysis

Consistent with our clinical choice and characteristics of model library, RapidPlan results of 10X photon were used as references, against which the performances of other beam energies were evaluated. Target homogeneity index (HI) was calculated as (*D*_2%_−*D*_98%_)/*D*_50%_, and conformity index (CI) was defined according to Paddick, et al [21]. D_x%_ indicates the minimum dose received by x% of the volume. In addition to conventional OARs such as urinary bladder, femoral heads and small bowel, the normal tissue integral dose (NTID) was calculated for the in-field body volume excluding PTV, and the in-field skin dose was calculated in the volume 5 mm under the body surface in accordance with previous dosiemtric studies on photon energies[4,10]. Normality test was first performed to the results of each group, followed by two-way ANOVA (repeated measures on the same individual without replication in each subgroup) and multiple paired T test (with significant level adjusted to 0.016) for further comparison if AVOVA tests reported P<0.05 (statistically significant, right-tailed).

## Results

### Patient characteristics

Table 1 summarizes the tumor characteristics of 81 training cases (53 males) and 20 validation cases (14 males). The ranges and medium age of the training set were 39~89 and 62 year, and were 34~75 and 60 year for the validation set respectively. The concurrent chemotherapy regimen was capecitabine 825mg/m^2^ twice daily, 5d/w.

**Table 1:** Tumor volumes [cm^3^] of 81 training cases and 20 validation cases.

	PTV_boost_		PTV	
	Training	Validation	Training	Validation
Mean	179.54	170.70	1207.99	1151.71
Standard deviation	92.00	96.85	165.91	187.73
Minimum	53.31	60.5	844.12	802.40
Maximum	618.09	478.2	1675.05	1562.00

### Target dose coverage using various photon energies

**Fig 1:**
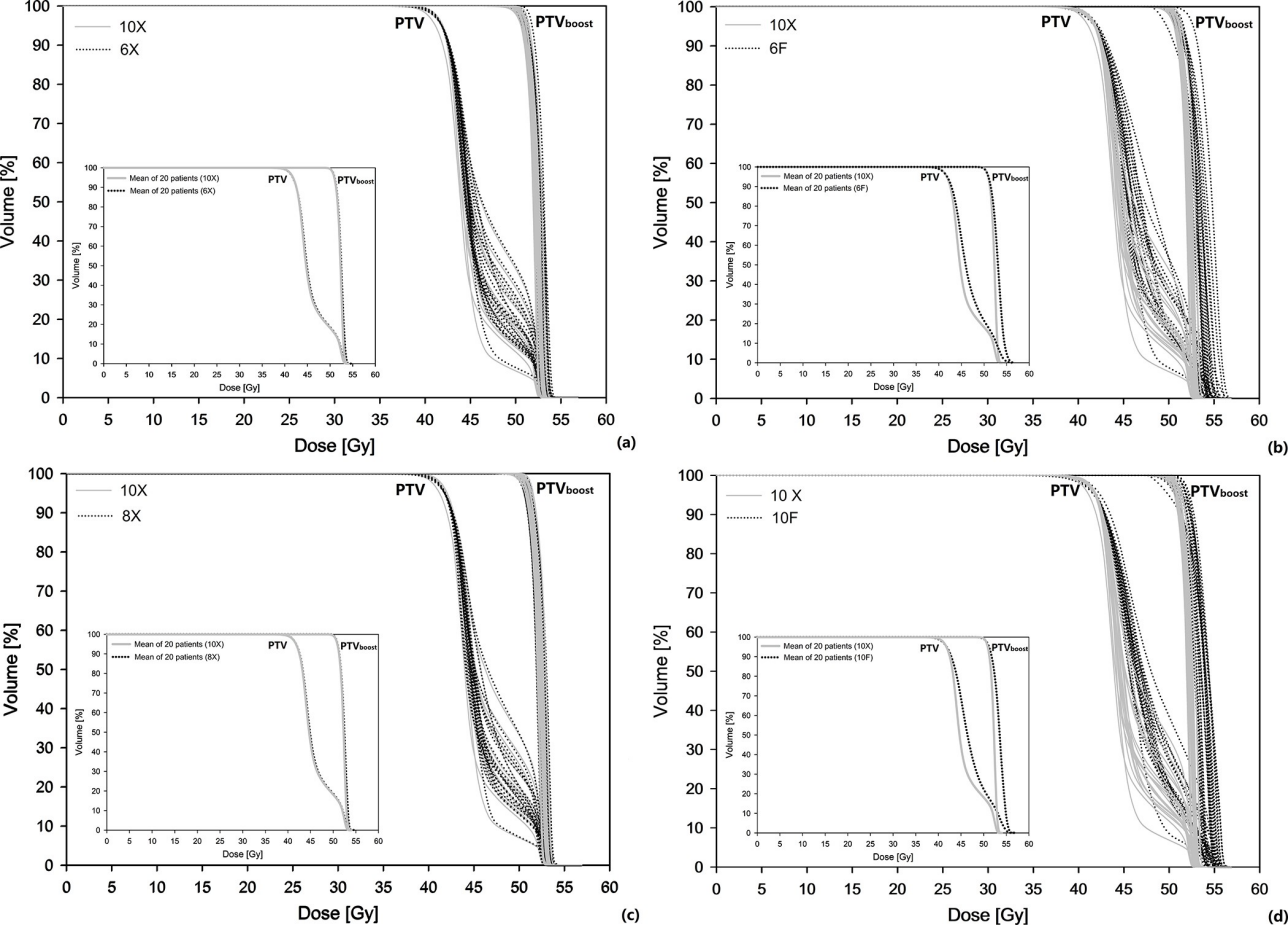
The target DVHs of 20 patients, as optimized automatically using various photon energies. Subfigures (a-d) present the target DVHs of 6X, 6F, 8X and 10 F (dotted lines) relative to the reference 10X results (solid lines) respectively. The figure-in-figure shows the corresponding mean target DVHs of 20 patients, as optimized using the same beam energy.

Fig 1 plots the target DVHs of 20 patients, as optimized automatically using various photon energies. Subfigures (a-d) present the target DVHs of 6X, 6F, 8X and 10 F (dotted lines) relative to the reference 10X results (solid lines) respectively. The figure-in-figure shows the corresponding mean target DVHs of 20 patients, as optimized using the same beam energy. The target DVHs of 10X, 8X and 6X largely overlapped with each other, providing comparable basis for OAR dose evaluation. Echoing numerically, the mean CI values for 6X, 8X and 10X were 1.05, 1.05, 1.04 for planning target volume (PTV), and 1.14, 1.12, 1.10 for PTV_boost_, respectively. The corresponding mean HI values for all flattened beams were 0.27 (PTV) and 0.05 (PTV_boost_) respectively. However, the dosimetric features such as dose falloff and hot spots were severely worsened using 6F and 10 F beams, making those plans clinically unacceptable which were thus excluded from further OAR dose comparison. Specifically, the mean CI values for 6F and 10F were 1.10, 1.10 (PTV), and 1.29, 1.34 (PTV_boost_) respectively. The corresponding mean HI values for 6F and 10F were 0.29, 0.30 (PTV) and 0.07, 0.08 (PTV_boost_) respectively.

### Impact of photon energies on normal tissue sparing

The dosimetric statistics of OARs comparing the performance of 6X and 8X using 10X results as reference were listed in Table 2.

**Table 2:** Dosimetric statistics of OARs comparing the results of 6X and 8X against 10X.

		6X	10X		8X
Urinary bladder					
	V_40Gy_	14.19	13.37		13.98
	V_45Gy_	3.23	3.24		3.10
	D_mean_	24.30	22.86		23.68
	P	<0.01		<0.01	
Femoral heads					
	V_40Gy_	0	0.01		0
	V_45Gy_	0	0		0
	D_mean_	13.58	13.00		13.40
	P	0.32		0.50	
NTID					
	D_mean_	17.92	17.27		17.41
	P	<0.01		0.02	
Skin					
	D_mean_	10.80	9.66		9.99
	P	<0.01		<0.01	
Small bowel					
	V_35Gy_	7.10	5.52		6.61
	V_40Gy_	1.09	0.67		1.01
	V_45Gy_	0	0		0
	D_mean_	22.78	21.42		22.28
	P	<0.01		<0.01	

P values were calculated for the mean dose. The units for the volume and dose are % and Gy respectively. Abbreviations: V_xGy_ = volume receiving at least x Gy dose; D_mean_ = mean dose; NTID = normal tissue integral dose.

**Fig 2:**
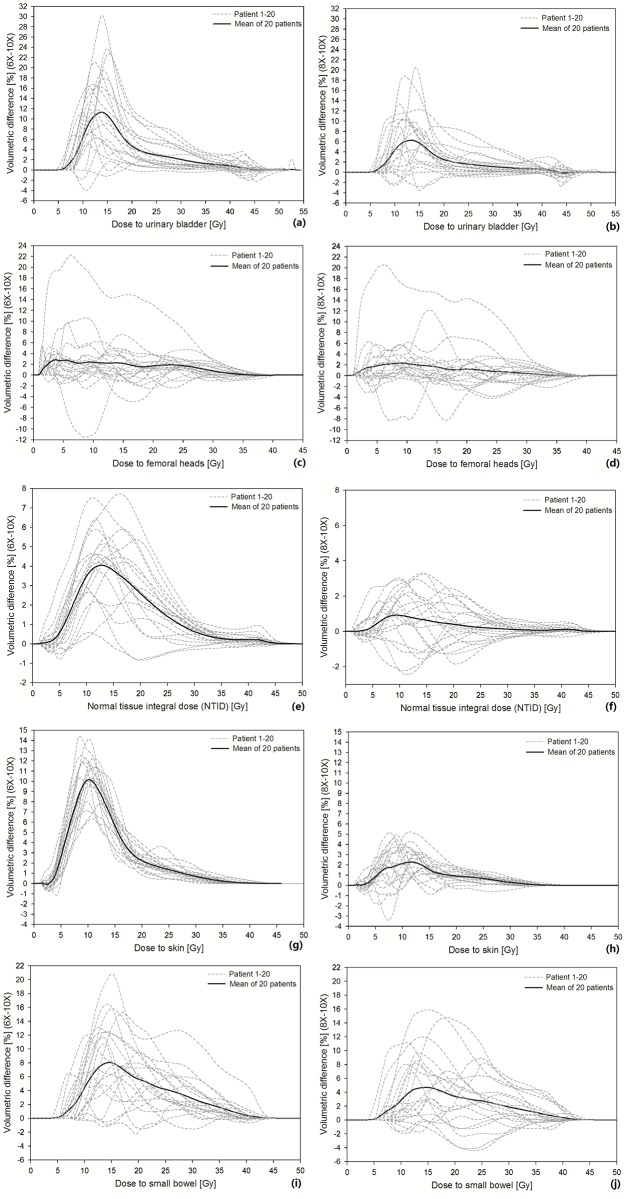
The DVH differences of the OARs as optimized using various beam energies relative to 10X photons. The dashed lines plot the data of 20 patients, and the solid lines are their average. The left and right column shows the results of 6X-10X and 8X-10X respectively. Subfigures (a-b), (c-d), (e-f), (g-h) and (i-j) plot the results for the urinary bladder, femoral heads, NTID, skin and small bowel respectively.

Fig 2 displays the DVH differences of the OARs as optimized using various beam energies relative to 10X photons. The differences were calculated by subtracting the DVHs of evaluated energy from the DVHs of 10X, hence curves above zero horizontal levels indicate worsened OAR sparing than those using 10X beams. The dashed lines plot the data of 20 patients, and the solid lines are their average. The left and right column shows the results of 6X-10X and 8X-10X respectively. Subfigures (a-b), (c-d), (e-f), (g-h) and (i-j) plot the results for the urinary bladder, femoral heads, NTID, skin and small bowel respectively.

## Discussion

According to the manufacturer, RapidPlan does not estimate DVHs for the targets, which is determined by the fixed objectives as imbedded in the model template. It seems that these parameters can be shared by flattened beams of various energies, yielding comparable target dose coverage as suggested by the largely overlapping DVHs in Fig 1. These observations agreed with the reports that satisfactory deep-seated target coverage can be achieved by using lower photon energies if more fields were used [5]. Dramatically differed in beam characteristics, these settings did not work well for un-flattened beams of either 6F or 10F. The insufficient dose coverage induced unacceptable hot spot after renormalization (severely worsened CI for PTV_boost_), suggesting that new optimization objectives for target dose should be configured before the RapidPlan model can be possibly used to optimize the modulation of un-flattened beams.

Although tremendous inter-patient varieties were observed for all OARs and beam energies in Fig 2, a majority of individual OAR curves and all the mean curves were overwhelmingly above zero, suggesting inferior OAR sparing using 6X and 8X than 10X. Excessive exposure was dominantly distributed in low dose regions (<20 Gy). The amplitudes of 8X curves were consistently lower than those of 6X, as echoed by the significantly higher dose to most OARs using lower beam energies in Table 2 (except for femoral heads, probably due to limited sample size), suggesting lower OAR exposures are associated with higher beam energies, at least for pelvic planning using a RapidPlan model configured with historical plans that were optimized using 10X photons. Individual lines of 8X showed more negative values and larger fluctuations than those of 6X, suggesting other factors started to play relatively more important roles in determining the OAR dose, when the energy difference reduces. These differences might be jointly contributed by multiple resources, such as beam energy (*λ*_*f*_) in the modeling of *ged*_*v*_, inherent photon behaviors, patient and beam geometries, etc., which can be hardly differentiated from each other. Due to relatively low values, the disparities of high dose volumes in Table 2 did not vary too much.

It should be noted that the skin and NTID are not typical OARs for rectal planning, hence were not configured in the RapidPlan model. They were analyzed in this work as indicators of superficial dose and normal tissue sparing in accordance with previous studies on beam energies[4,10]. Classical radiotherapy principle recommended higher beam energy for deep seated large tumors [1], but lower energy can reduce exit dose [22] and Pirzkall et al reported negligible difference among different energies when more than 9 IMRT fields were used [5]. In the context of knowledge-based planning, our results suggested that higher beam energies were still advantageous in normal tissue sparing for deep seated tumors of large volumes, even if many beam entries were used such as VMAT technique. Without potential bias from manual adjustments, the RapidPlan-generated optimization objectives assessed the beam quality collectively with other influential parameters such as patient anatomy and beam geometry in Eq (1), hence the dosimetric comparison between various beam energies were more objective.

## Conclusion

In conclusion, a RapidPlan model configured with flattened high energy beams does not satisfy target dose coverage using un-flattened photons, and may increase normal tissue exposure if applied to optimize lower energy beams.

## References

[1] Laughlin JS, Mohan R, Kutcher GJ. Choice of optimum megavoltage for accelerators for photon beam treatment. International Journal of Radiation Oncology• Biology• Physics. 1986 9 1;12(9):1551–7. 10.1016/0360-3016(86)90277-4 3093417

[2] Welsh JS, Mackie TR, Limmer JP. High-energy photons in IMRT: uncertainties and risks for questionable gain. Technology in cancer research & treatment. 2007 4;6(2):147–9. 17375978 10.1177/153303460700600212

[3] Ost P, Speleers B, De Meerleer G, De Neve W, Fonteyne V, Villeirs G, et al Volumetric arc therapy and intensity-modulated radiotherapy for primary prostate radiotherapy with simultaneous integrated boost to intraprostatic lesion with 6 and 18 MV: a planning comparison study. International Journal of Radiation Oncology* Biology* Physics. 2011 3 1;79(3):920–6. 10.1016/j.ijrobp.2010.04.025 20675077

[4] Yadav G, Bhushan M, Dewan A, Saxena U, Kumar L, Chauhan D, et al Dosimetric influence of photon beam energy and number of arcs on volumetric modulated arc therapy in carcinoma cervix: A planning study. Reports of Practical Oncology & Radiotherapy. 2017 1 1;22(1):1–9. 27790072 10.1016/j.rpor.2016.09.002 PMC5071544

[5] Pirzkall A, Carol MP, Pickett B, Xia P, Roach M III, Verhey LJ. The effect of beam energy and number of fields on photon-based IMRT for deep-seated targets. International Journal of Radiation Oncology* Biology* Physics. 2002 6 1;53(2):434–42. 10.1016/s0360-3016(02)02750-5 12023148

[6] Yuan L, Ge Y, Lee WR, Yin FF, Kirkpatrick JP, Wu QJ. Quantitative analysis of the factors which affect the interpatient organ‐at‐risk dose sparing variation in IMRT plans. Medical physics. 2012 11;39(11):6868–78. 10.1118/1.4757927 23127079

[7] Shiraishi S, Moore KL. Knowledge‐based prediction of three‐dimensional dose distributions for external beam radiotherapy. Medical physics. 2016 1 1;43(1):378–87. 10.1118/1.4938583 26745931

[8] Varian Medical Systems 2015 Eclipse Photon and Electron Algorithms Reference Guide P1008611-003-C 226–227

[9] Onal C, Sonmez S, Erbay G, Guler OC, Arslan G. Simultaneous integrated boost to intraprostatic lesions using different energy levels of intensity-modulated radiotherapy and volumetric-arc therapy. The British journal of radiology. 2014 1 8;87(1034):20130617 10.1259/bjr.20130617 24319009 PMC4064542

[10] Mattes MD, Tai C, Lee A, Ashamalla H, Lkoro NC.The dosimetric effects of photon energy on the quality of prostate volumetric modulated arc therapy. Pract Radiat Oncol 2014;4(1): e39 10.1016/j.prro.2013.03.001 24621430

[11] Wu H, Jiang F, Yue H, Li S, Zhang Y. A dosimetric evaluation of knowledge‐based VMAT planning with simultaneous integrated boosting for rectal cancer patients. Journal of applied clinical medical physics. 2016 11 1;17(6):78–85. 10.1120/jacmp.v17i6.6410 27929483 PMC5690500

[12] Wu H, Jiang F, Yue H, Zhang H, Wang K, Zhang Y. Applying a RapidPlan model trained on a technique and orientation to another: a feasibility and dosimetric evaluation. Radiation Oncology. 2016 12;11(1):108 10.1186/s13014-016-0684-9 27538431 PMC4990878

[13] Jiang F, Wu H, Yue H, Jia F, Zhang Y. Photon Optimizer (PO) prevails over Progressive Resolution Optimizer (PRO) for VMAT planning with or without knowledge‐based solution. Journal of applied clinical medical physics. 2017 3;18(2):9–14. 10.1002/acm2.12038 28300375 PMC5689948

[14] Li JL, Ji JF, Cai Y, Li XF, Li YH, Wu H, et al Preoperative concomitant boost intensity-modulated radiotherapy with oral capecitabine in locally advanced mid-low rectal cancer: a phase II trial. Radiotherapy and Oncology. 2012 1 1;102(1):4–9. 10.1016/j.radonc.2011.07.030 21903285

[15] Hong TS, Moughan J, Garofalo MC, Bendell J, Berger AC, Oldenburg NB, et al NRG Oncology Radiation Therapy Oncology Group 0822: a phase 2 study of preoperative chemoradiation therapy using intensity modulated radiation therapy in combination with capecitabine and oxaliplatin for patients with locally advanced rectal cancer. International Journal of Radiation Oncology* Biology* Physics. 2015 9 1;93(1):29–36. 10.1016/j.ijrobp.2015.05.005 PMC4540628 26163334

[16] Cheung MR, Tucker SL, Dong L, De Crevoisier R, Lee AK, Frank S, et al Investigation of bladder dose and volume factors influencing late urinary toxicity after external beam radiotherapy for prostate cancer. International Journal of Radiation Oncology* Biology* Physics. 2007 3 15;67(4):1059–65. 10.1016/j.ijrobp.2006.10.042 PMC2081969 17241755

[17] Heemsbergen WD, Hoogeman MS, Hart GA, Lebesque JV, Koper PC. Gastrointestinal toxicity and its relation to dose distributions in the anorectal region of prostate cancer patients treated with radiotherapy. International Journal of Radiation Oncology* Biology* Physics. 2005 3 15;61(4):1011–8. 10.1016/j.ijrobp.2004.07.724 15752880

[18] Boice JD Jr, Day NE, Andersen A, Brinton LA, Brown R, Choi NW, et al Second cancers following radiation treatment for cervical cancer. An international collaboration among cancer registries. Journal of the National Cancer Institute. 1985 5 1;74(5):955–75. 3858584

[19] Griem ML. Neutron Contamination From Medical Electron Accelerators. JAMA. 1985 12 6;254(21):3108–9.

[20] Wu B, Ricchetti F, Sanguineti G, Kazhdan M, Simari P, Jacques R, et al Data-driven approach to generating achievable dose–volume histogram objectives in intensity-modulated radiotherapy planning. International Journal of Radiation Oncology* Biology* Physics. 2011 3 15;79(4):1241–7. 10.1016/j.ijrobp.2010.05.026 20800382

[21] Paddick I. A simple scoring ratio to index the conformity of radiosurgical treatment plans. Journal of neurosurgery. 2000 12;93(Supplement 3):219–22. 11143252 10.3171/jns.2000.93.supplement

[22] Sun M., & Ma L. (2006). Treatments of exceptionally large prostate cancer patients with low-energy intensity-modulated photons. Journal of Applied Clinical Medical Physics, 7(4), 43–49. 10.1120/jacmp.v7i4.2263 17533352 PMC5722392

